# Editorial: Critical complications in pediatric oncology and hematopoietic cell transplant, volume II

**DOI:** 10.3389/fonc.2024.1512659

**Published:** 2024-11-01

**Authors:** Jennifer Ann McArthur, Kris M. Mahadeo, Asya Agulnik, Marie E. Steiner

**Affiliations:** ^1^ Division of Critical Care Medicine, Department of Pediatrics, St Jude Children’s Research Hospital, Memphis, TN, United States; ^2^ Division of Pediatric Transplantation and Cellular Therapy, Duke University School of Medicine, Durham, NC, United States; ^3^ Department of Pediatric Global Medicine, St Jude Children’s Research Hospital, Memphis, TN, United States; ^4^ Division of Pediatric Hematology Oncology, M Health Fairview Masonic Children’s Hospital, Minneapolis, MN, United States

**Keywords:** pediatric cancer, pediatric critical care, hematopoietic cell transplant, pediatric oncology and hematology, multi-disciplinary communication, early recognition

## Summary of volume 1

In the early years, mortality rates for pediatric hematopoietic cell transplant (HCT) patients with critical illness were abysmal, exceeding 80%. This led to the general belief that providing critical care resources to this population was futile (1). Volume I of this Research Topic published 30 articles from 211 authors in 9 different countries (2). In this first volume, Pechlaner et al. reported a PICU mortality of 11% for pediatric hematology/oncology patients (3) – a significant improvement from the early years. This volume extensively discussed management of complications from HCT, cancer, and chimeric antigen receptor therapy (CAR-T) (4–6). Management of these complications involved utilization of critical care resources such as continuous renal replacement therapy (CRRT) (7), extracorporeal membrane oxygenation (ECMO) (8), and mechanical ventilation (9) – resources that would not have been considered for this population in the early years.

Improvement in outcomes may be partially explained by topics discussed in this first volume. These include 1) utilization of strategies to promote early recognition of clinical deterioration leading to earlier interventions and involvement of critical care teams (5, 9–13); 2) use of invasive diagnostic procedures such as bronchial alveolar lavage and lung biopsy which may lead to more accurate diagnoses and targeted therapies (14, 15); and 3) careful attention to detail such as prevention of the detrimental effects of fluid overload (16).

**Figure 1:**
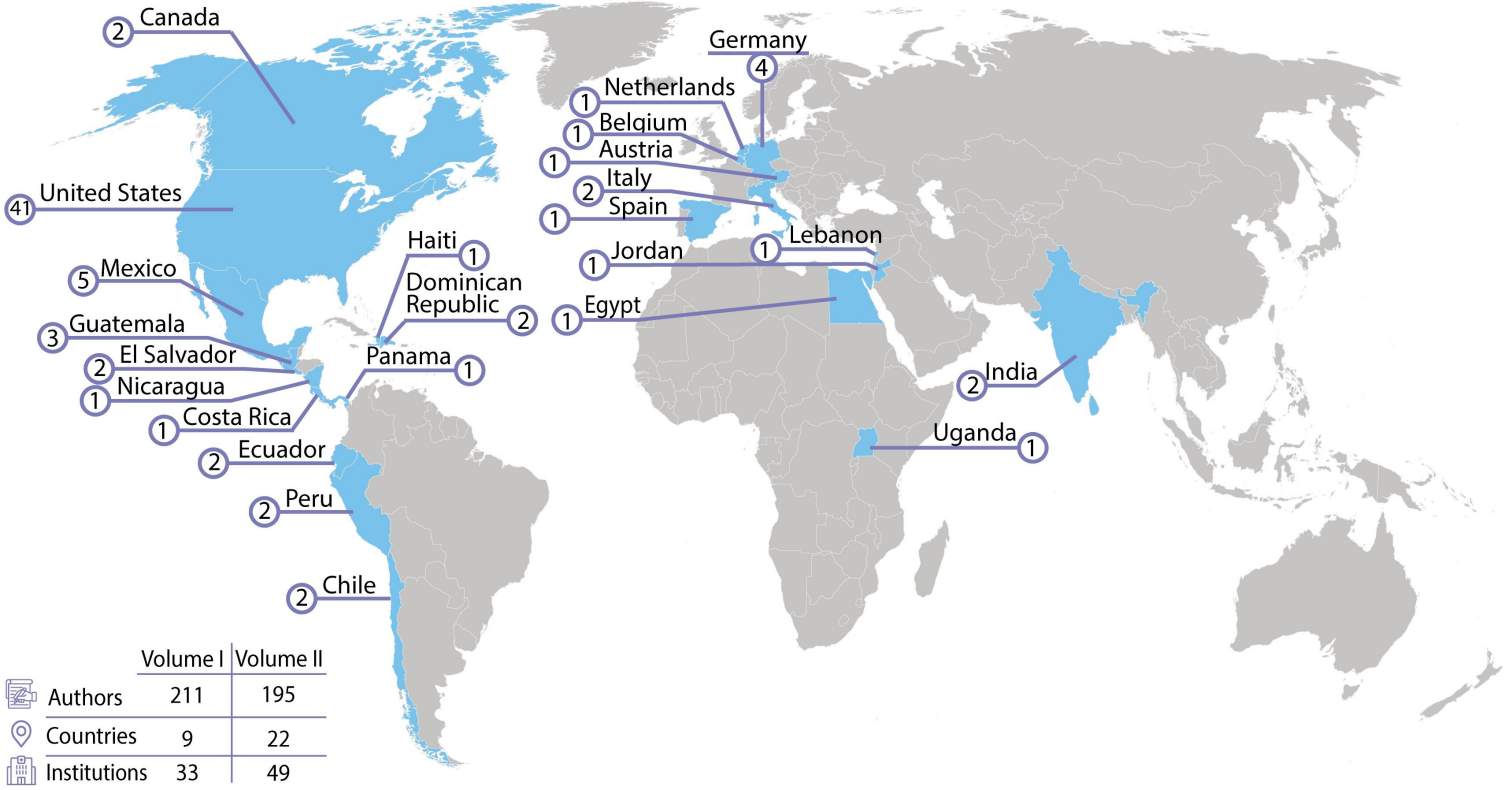
Evidence for growing international interest in the field of pediatric onco-critical care.

In the current Research Topic, Critical Complications in Pediatric Oncology and Hematopoietic Cell Transplant, Volume II, there is a continuation of the themes of improving outcomes and strengthening collaboration. This Research Topic contains 21 publications from 195 authors representing 22 different countries on 5 continents (**Figure 1**). Volume II provides ongoing evidence that the field of pediatric onco-critical care is not going back to the era of the self-fulfilling prophecy that critically ill children with cancer have abysmal outcomes rendering use of critical care resources futile.

## Predictive factors for critical care needs

Knowing which HCT patients are at highest risk for requiring ICU care would be very valuable for clinicians. Using data from pediatric oncology patients in the Colorado Sepsis and Treatment Registry, serum lactate within 2 hours of presentation was found to be predictive of clinical deterioration events (OR 1.82, p<0.001), need for ICU admission (OR 1.68, p<0.001) and bacteremia (OR 1.49, p<0.001) (Slatnick et al.). Johnson et al. performed a single center retrospective review of pediatric patients who received HCT at their institution between January 2015-December 2020. Risk factors for PICU admission were: 1) younger age; 2) lower weight; 3) inborn error of metabolism as a reason for HCT and 4) use of busulfan conditioning. There was overlap in these results with those found by Zinter et al. in a multi-center study merging the Center for International Bone Marrow Transplantation (CIBMTR) and Virtual PICU Performance System (VPS) databases. They also found younger age and inborn errors of metabolism as risk factors for requiring ICU care (17). However, there was disagreement where Zinter found pre-HCT organ dysfunction was associated with increased requirement for ICU admission, whereas Johnson did not. This may represent an improvement over time in managing complex patients during HCT versus differences in study design. A better understand organ dysfunction in these unique patients is imperative for continued improvements in outcomes.

## PICU resource utilizations and outcomes

Accurate data surrounding the risks and benefits of ICU therapies will lead to better informed decisions regarding PICU interventions. In a retrospective single center study, Schober et al. found that admissions for respiratory support (OR 1.04, p=0.04) and dialysis (OR 1.21, p=0.03) increased 6-month mortality compared to other reasons for PICU admission. In a multi-variate analysis of pediatric oncology patients, hemato-oncology diagnosis, number of failing organs at baseline and unplanned admissions were associated with development of new or progressive multi-organ failure (Soeteman et al.). Data from the Health Facts (Cerner Corporation, Kansas City, MO) database containing 473 pediatric HCT patients found 11% required positive pressure ventilation, 25% received vasopressor medications and 3% received dialysis. Decreased survival was seen in allogeneic transplant (p<0.01), graft versus host disease (p=0.02), infection (p<0.01) and need for ICU therapies (p<0.01) (Olson et al.). Interestingly, survival improved over time for patients who received allogeneic transplants. The improved survival in the later era of the study was associated with decreased infections and increased use of vasopressor agents. The improvement in survival could represent a change in practice due to recent publications addressing the detrimental effects of fluid overload in HCT patients (16, 18) with a shift towards earlier use of vasopressors rather than fluid resuscitation.

Chimeric antigen receptor therapy, CAR-T, is being used in a growing number of cancers. However, it carries an increased risk for life threatening complications and critical illness. In a multi-center study, Ragoonanan et al. compared PICU courses for pediatric ALL patients who were receiving conventional therapy vs those who received tisagenlecleucel. They found PICU resource utilization between the 2 groups to be similar. The authors concluded that improved management of complications and need for ICU care should decline over time making CAR-T an important therapy to pursue, potentially beyond high resource settings.

Cardenas-Aguirre et al. show us that critically ill pediatric oncology patients in resource limited-settings can have PICU outcomes similar to those seen in high income countries. In their dedicated pediatric oncology hospital in Mexico, they describe overall PICU mortality of 6.9% with mortality for unplanned PICU admissions of 9.1%. This is similar to that described in high income countries (19, 20) The authors felt their center’s low mortality was likely the result of implementing a number of quality improvement practices aimed at earlier recognition of deterioration allowing for earlier interventions.

## Complications of HCT and oncology therapy

Endotheliopathy has been considered an underlying cause of multiple complications of HCT including sinusoidal obstructive disorder (SOS), transplant associated – thrombotic microangiopathy (TA-TMA), diffuse alveolar hemorrhage (DAH), pulmonary hypertension and graft-versus-host disease (GVHD). In a review article, Pace et al. explore the interaction between host and donor endothelial cells in hematopoietic cell transplantation as well as solid organ transplant. Kafa et al. described their single center experience with TA-TMA. Factors associated with developing TA-TMA were allogeneic transplant and use of total body irradiation as part of the conditioning regimen. Despite a good response to therapy, their patients experienced several complications with the most frequent being renal impairment and chronic kidney disease in 80%.

This Research Topic also addresses strategies for improving management of respiratory failure, a deadly complication. Pediatric HCT patients have been shown to have a high rate of peri-intubation cardiac arrest (9) which may represent a delay in intubation timing. Hume et al. undertook a survey of PICU and HCT providers to understand beliefs around timing of intubation. Clinicians agreed that a patient’s poor prognosis may delayed their decision to intubate. However, their decision was not influenced by increased risk for lung injury from prolonged non-invasive intubation and/or oxygen, factors likely to be important (21–23).

DAH after HCT has historically had high mortality rates (24–27). Our Research Topic has two retrospective chart review studies discussing novel therapies in DAH. In a multi-center study, there was an increased risk of non-relapse mortality with use of steroids (p=0.03), once considered standard therapy for DAH, and a survival advantage with use of inhalation of tranexamic acid (p=0.04) or recombinant activated factor VII (p=0.005) (Schoettler et al.). A single center study confirmed the safety of inhaled recombinant activated Factor VII for management of DAH in these patients (Hurley et al.).

Pulmonary hypertension (PH) is yet another complication of cancer treatment and HCT thought to be related to endothelial injury. An analysis of merged Center for International Blood and Marrow Transplant Research (CIBMTR) and the Virtual Pediatric System (VPS) databases showed a PH prevalence of 2.7% in pediatric HCT patients requiring ICU care. Of patients with PH admitted to the PICU, 72.4% required invasive mechanical ventilation and 27.6% renal replacement therapies. Survival 6 months after PH diagnosis was 51.7%, making this a very deadly disease lacking effective therapy (Smith et al.).

Renal failure as a complication of HCT is common and known to be a strong predictor of mortality (28, 29). Vuong et al. reviewed the available published data on acute kidney injury and chronic kidney disease in patients post-HCT. This review points out the importance of early identification of renal dysfunction enabling timely interventions to decrease risk of progression to end stage renal disease. Anderson et al. performed a single center retrospective chart review study of 222 pediatric oncology patients admitted for tumor lysis syndrome. They discovered 9% of patients with tumor lysis syndrome required renal replacement therapy (RRT), most commonly for metabolic abnormalities. All patients with tumor lysis syndrome survived to hospital discharge and none required chronic renal support. The experience for RRT in patients with tumor lysis differs significantly from the experience in patients post-HCT.

Cytomegalovirus (CMV) is one of the most concerning infections for patients post-transplant. Many patients go into their transplant course with latent infections which may reactivate during periods of immunosuppression. Hiskey et al. provide us with an excellent review of strategies for prevention, early detection, and intervention to mitigate the impact of CMV in these patients.

## Multidisciplinary care and communication

In Volume I, Agulnik et al. demonstrated that implementation of a bedside pediatric early warning system (PEWS) led to earlier recognition of critical illness and prompt interventions (12). In Volume II, Abutineh et al. describe the implementation of PEWS at 23 pediatric cancer centers across Latin America. The authors found that resources were important in enabling the adaptation and implementation of PEWS in these settings. Prior experience of the hospital or its leaders with quality improvement (QI), however, was helpful for overcoming the inevitable challenges involved in implementing PEWS. In the absence of prior QI experience, QI training was also helpful. Mirochnik et al. analyzed 71 structured interviews with clinical staff in 5 resource limited pediatric oncology centers in Latin America. Interviewed clinicians described PEWS as making them feel more knowledgeable, confident, and empowered in their patient care duties leading to improved job satisfaction and patient outcomes.

Rivera et al. described the development of a first-in-kind tool to measure the quality of multi-disciplinary and interprofessional communication around clinical deterioration in children with cancer. Their tool, CritCom, was developed through literature review and use of a multidisciplinary panel of experts. A later publication from Counts et al. discussed the process involved in refining the CritCom reports given to centers to communicate CritCom findings and allow their use for local QI. This process can be utilized by other groups wanting to improve communication of research/QI findings to stakeholders.

Cuviello et al. discuss the importance of interdisciplinary communication during end-of-life care. They performed a retrospective chart review study involving 43 pediatric oncology patients receiving end-of-life care in the PICU. They found 18.6% of patients did not have palliative care involvement until the day of death and that almost half of patients were receiving cancer directed therapy in their last week of life. Their findings suggest room for improvement through earlier collaboration between the palliative care, oncology, and ICU teams.

## Future of onco-critical care

Critical care resources for critically ill pediatric oncology, and HCT patients in high resource settings is clearly no longer futile. Patients are now routinely offered aggressive supportive care measures with improving survival and reduced morbidity. We are not going back to the days of the self-fulfilling prophecy that these patients have poor outcomes making PICU care futile. We look to the future as we progress towards improving outcomes globally, especially in limited resource settings where 90% of children with cancer reside.

Some of the most promising strategies to improve outcomes are aimed at early recognition of clinical deterioration enabling earlier interventions. These strategies can be implemented successfully in lower-resource settings as has been discussed in both volumes of this Research Topic. An additional advantage of implementing these systems is that they can improve multi-disciplinary and multiprofessional communication leading to improved job satisfaction and better patient care.

Improved understanding of the pathophysiologic mechanisms behind complications of HCT and cancer therapies will lead us toward more specific and effective novel therapies. We are just beginning to understand all the functions of the endothelium and what can go wrong when it is damaged. Next generation cancer therapies will include expansion of the scope of CAR-T and other targeted therapies. These therapies aim to harness the patient’s immune system to attack the cancer but incur risk of life-threatening complications. In the future, we expect improved therapies specifically targeting side effects while maintaining anti-cancer activity. The future of the field of onco-critical care is bright as we collaborate globally to achieve better outcomes for critically ill children with cancer worldwide.

## References

[1] Denardo SJ Oye RK Bellamy PE. Efficacy of intensive care for bone marrow transplant patients with respiratory failure. Crit Care Med. (1989) 17:4–6. doi: 10.1097/00003246-1989010000-00002 2642401

[2] Agulnik A Mahadeo KM Steiner ME McArthur JA. Editorial: Critical complications in pediatric oncology and hematopoietic cell transplant - how far we have come and how much further we must go. Front Oncol. (2023) 13:1148321. doi: 10.3389/fonc.2023.1148321 36910613 PMC9992885

[3] Pechlaner A Kropshofer G Crazzolara R Hetzer B Pechlaner R Cortina G. Mortality of hemato-oncologic patients admitted to a pediatric intensive care unit: A single-center experience. Front Pediatr. (2022) 10:795158. doi: 10.3389/fped.2022.795158 35903160 PMC9315049

[4] Baumeister SHC Mohan GS Elhaddad A Lehmann L. Cytokine release syndrome and associated acute toxicities in pediatric patients undergoing immune effector cell therapy or hematopoietic cell transplantation. Front Oncol. (2022) 12:841117. doi: 10.3389/fonc.2022.841117 35402259 PMC8989409

[5] Brown BD Tambaro FP Kohorst M Chi L Mahadeo KM Tewari P. Immune effector cell associated neurotoxicity (ICANS) in pediatric and young adult patients following chimeric antigen receptor (CAR) T-cell therapy: can we optimize early diagnosis? Front Oncol. (2021) 11:634445. doi: 10.3389/fonc.2021.634445 33763368 PMC7982581

[6] Harden A Ragoonanan D Anildes-Gubman D McCall D Faltus K Featherston S. Chimeric antigen receptor, teamwork, education, assessment, and management (CAR-TEAM): A simulation-based inter-professional education (IPE) intervention for management of CAR toxicities. Front Oncol. (2020) 10:1227. doi: 10.3389/fonc.2020.01227 32850365 PMC7419673

[7] Elbahlawan L Bissler J Morrison RR. Continuous renal replacement therapy: A review of use and application in pediatric hematopoietic stem cell transplant recipients. Front Oncol. (2021) 11:632263. doi: 10.3389/fonc.2021.632263 33718216 PMC7953134

[8] Ghafoor S Fan K Di Nardo M Talleur AC Saini A Potera RM. Extracorporeal membrane oxygenation candidacy in pediatric patients treated with hematopoietic stem cell transplant and chimeric antigen receptor T-cell therapy: an international survey. Front Oncol. (2021) 11:798236. doi: 10.3389/fonc.2021.798236 35004323 PMC8727600

[9] Rowan CM Fitzgerald JC Agulnik A Zinter MS Sharron MP Slaven JE. Risk factors for noninvasive ventilation failure in children post-hematopoietic cell transplant. Front Oncol. (2021) 11:653607. doi: 10.3389/fonc.2021.653607 34123807 PMC8190382

[10] Graetz DE Giannars E Kaye EC Garza M Ferrara G Rodriguez M. Clinician emotions surrounding pediatric oncology patient deterioration. Front Oncol. (2021) 11:626457. doi: 10.3389/fonc.2021.626457 33718195 PMC7947818

[11] Garza M Graetz DE Kaye EC Ferrara G Rodriguez M Soberanis Vásquez DJ. Impact of PEWS on perceived quality of care during deterioration in children with cancer hospitalized in different resource-settings. Front Oncol. (2021) 11:660051. doi: 10.3389/fonc.2021.660051 34249696 PMC8260684

[12] Agulnik A Gossett J Carrillo AK Kang G Morrison RR. Abnormal vital signs predict critical deterioration in hospitalized pediatric hematology-oncology and post-hematopoietic cell transplant patients. Front Oncol. (2020) 10:354. doi: 10.3389/fonc.2020.00354 32266139 PMC7105633

[13] Traube C Gerber LM Mauer EA Small K Broglie L Chopra YR. Delirium in children undergoing hematopoietic cell transplantation: A multi-institutional point prevalence study. Front Oncol. (2021) 11:627726. doi: 10.3389/fonc.2021.627726 33968727 PMC8100670

[14] Ahmad AH Brown BD Andersen CR Mahadeo KM Petropolous D Cortes JA. Retrospective review of flexible bronchoscopy in pediatric cancer patients. Front Oncol. (2021) 11:770523. doi: 10.3389/fonc.2021.770523 34970488 PMC8712312

[15] Elbahlawan L McArthur J Morin CE Abdelhafeez H McCarville MB Ruiz RE. Pulmonary complications in children following hematopoietic cell transplantation: A case report and review of the diagnostic approach. Front Oncol. (2021) 11:772411. doi: 10.3389/fonc.2021.772411 34820335 PMC8606675

[16] Sallee CJ Smith LS Rowan CM Heckbert SR Angelo JR Daniel MC. Early cumulative fluid balance and outcomes in pediatric allogeneic hematopoietic cell transplant recipients with acute respiratory failure: A multicenter study. Front Oncol. (2021) 11:705602. doi: 10.3389/fonc.2021.705602 34354951 PMC8329703

[17] Zinter MS Brazauskas R Strom J Chen S Bo-Subait S Sharma A. Critical illness risk and long-term outcomes following intensive care in pediatric hematopoietic cell transplant recipients. Blood Adv. (2024) 8:1002–17. doi: 10.1182/bloodadvances.2023011002 PMC10879681 38127268

[18] Agarwal N Rotz S Hanna R. Medical emergencies in pediatric blood & marrow transplant and cellular therapies. Front Pediatr. (2023) 11:1075644. doi: 10.3389/fped.2023.1075644 36824648 PMC9941678

[19] Zinter MS DuBois SG Spicer A Matthay K Sapru A. Pediatric cancer type predicts infection rate, need for critical care intervention, and mortality in the pediatric intensive care unit. Intensive Care Med. (2014) 40:1536–44. doi: 10.1007/s00134-014-3389-2 PMC4177269 25023526

[20] Rr P Tan EEK Sultana R Thoon KC Chan MY Lee JH. Critical illness epidemiology and mortality risk in pediatric oncology. Pediatr Blood Cancer. (2020) 67:e28242. doi: 10.1002/pbc.28242 32187445

[21] Cater DT Fitzgerald JC Gertz SJ McArthur JA Daniel MC Mahadeo KM. Noninvasive ventilation exposure prior to intubation in pediatric hematopoietic cell transplant recipients. Respir Care. (2022) 67:1121. doi: 10.4187/respcare.09776 35640999 PMC9994337

[22] Gertz SJ Bhalla A Chima RS Emeriaud G Fitzgerald JC Hsing DD. Immunocompromised-associated pediatric acute respiratory distress syndrome: experience from the 2016/2017 pediatric acute respiratory distress syndrome incidence and epidemiology prospective cohort study. Pediatr Crit Care Med. (2024) 25:288–300. doi: 10.1097/PCC.0000000000003421 38236083 PMC10994753

[23] Greico DL Maggiore SM Rocs O Spinelli E Patel BK Thille AW. Non-invasive ventilatory support and high-flow nasal oxygen as first-line treatment of hypoxemic respiratory failure and ARDS. Intensive Care Med. (2021) 47:851–66. doi: 10.1007/s00134-021-06459-2 PMC8261815 34232336

[24] Lewis ID DeFor T Weisdorf DJ. Increasing incidence of diffuse alveolar hemorrhage following allogeneic bone marrow transplantation: cryptic etiology and uncertain therapy. Bone Marrow Transplant. (2000) 26:539–43. doi: 10.1038/sj.bmt.1702546 11019844

[25] Robbins RA Linder J Stahl MG Thompson AB 3rd Haire W Kessinger A. Diffuse alveolar hemorrhage in autologous bone marrow transplant recipients. Am J Med. (1989) 87:511–8. doi: 10.1016/0002-9343(89)90690-6 2816966

[26] Afessa B Tefferi A Litzow MR Krowka MJ Wylam ME Peters SG. Diffuse alveolar hemorrhage in hematopoietic stem cell transplant recipients. Am J Respir Crit Care Med. (2002) 166:641–5. doi: 10.1164/rccm.200112-141CC 12204858

[27] Ben-Abraham R Paret G Cohen R Szold O Civdalli G Toren A. Diffuse alveolar hemorrhage following allogeneic bone marrow transplantation in children. Chest. (2003) 124:660–4. doi: 10.1378/chest.124.2.660 12907557

[28] Hingorani S. Renal complications of hematopoietic-cell transplantation. In: Ingelfinger JR, editor. New England Journal of Medicine, vol. 374 . Massachusetts Medical Society, Waltham, MA (2016). p. 2256–67. doi: 10.1056/NEJMral404711 27276563

[29] Bauer A Carlin K Schwartz SM Srikanthan M Thakar M Burroughs LM. Risk factors for severe acute kidney injury after pediatric hematopoietic cell transplantation. Pediatr Nephrol. (2022) 38:1365–72. doi: 10.1007/s00467-022-05731-x 36125547

